# Autopsy findings in COVID-19 infection-related death: a systematic review

**DOI:** 10.1186/s41935-022-00280-8

**Published:** 2022-05-10

**Authors:** Nik Muhammad Faiz Bin Nik Sofizan, Ahmad Faiz Bin Abd Rahman, Lai Poh Soon, Chng Kay Ly, Nor Zamzila Bt. Abdullah

**Affiliations:** 1 Kulliyyah of Medicine, International Islamic University Malaysia, 25200 Kuantan, Pahang Malaysia; 2 National Institute of Forensic Medicine (NIFM), Kuala Lumpur Hospital, Jalan Pahang, 50586 Kuala Lumpur, Wilayah Persekutuan Malaysia; 3 Forensic Unit, Pathology Department, Sultan Ahmad Shah Medical Centre @International Islamic University Malaysia, 25200 Jalan Sultan Haji Ahmad, Kuala Lumpur, Pahang Malaysia; 4 Pathology Department, Sultan Ahmad Shah Medical Centre @International Islamic University Malaysia, 25200 Jalan Sultan Haji Ahmad, Kuala Lumpur, Pahang Malaysia

**Keywords:** Post-mortem, Autopsy, COVID-19, Death

## Abstract

**Introduction:**

Coronavirus-19 disease (COVID-19) has been declared as pandemic by the World Health Organization (WHO) in March 2020. As of 28 November 2021, there were more than 260 million cases and nearly 5.2 million deaths caused by COVID-19. The most affected system by COVID-19 infection was the respiratory system although several other studies suggested multi-organ involvement with pathophysiology that was not clearly understood. Autopsy findings were beneficial to researchers to determine the mechanism behind these organ failures. The objective of this review was to summarize the autopsy findings related to COVID-19 death.

**Method:**

Online literature search was conducted via online databases such as Scopus, PubMed and Google Scholar. The keywords inputted during the search were “post-mortem”, “autopsy” and “COVID-19” in title, abstract and keywords. The inclusion criteria were the topic related with the title of this review, published in 2020–2021, have full text available and in English language. Any articles that were not related, duplicated studies, review articles including systematic review and meta-analysis and in other languages were excluded.

**Results:**

A total of 20 articles were included in this review. The articles reviewed were mostly case reports and case series while others were case-control and cohort study ranging from one to 348 cases. Majority were originated from the United States of America (USA).

**Conclusion:**

The most frequent system described in autopsy findings in COVID-19 death was the respiratory system, with the most common histological finding of diffuse alveolar damage (DAD). Majority of the findings of other organs were related to chronic diseases.

## Background

In early 2020, the world has been widely affected with newly found and highly infectious COVID-19. World Health Organization (WHO) has declared this infection as pandemic in March 2020.(World Health Organization (WHO) n.d.-a) The number of cases worldwide has been in ups-and-downs trend as the advancement of medical technology such as the production of COVID-19 vaccines and the newly discovered variants of COVID-19. Despite the procurement of vaccines around the world, WHO advised all the citizens to practice usage of face masks, hand hygiene and physical distancing as these were part of the strategy to curb COVID-19 infection. As of the end of November 2021, there were more than 260 million cases and approximately 5.2 million deaths have been reported globally.(World Health Organization (WHO) n.d.-b)

It has been clear that the most affected system by COVID-19 is the respiratory system although there were several studies that suggested multiple system involvement. COVID-19 infection could also lead to acute kidney injury, acute liver injury, cardiovascular diseases and haematological abnormalities. On top of that, people with comorbidities such as cardiovascular diseases, hypertension, obesity and others were at a higher risk for multiple system involvement.(Mokhtari et al. 2020; Zaim et al. 2020) However, the pathophysiology behind these organ failures was not well understood. Autopsy investigations of COVID-19 deaths were important to determine the cause of multi-organ failures which will subsequently help in proper management strategy for COVID-19 infection.

Thus, the aim for this article review was to collect and summarize the findings of autopsy of COVID-19 related deaths.

## Main text

The articles included must be related with the topic of this review, in English language, have full-text availability and be published from the year 2020 to 2021 which corresponds with the period of COVID-19 infection. Any articles that were not related with the topic, duplicated studies, review articles including systematic review and meta-analysis and in any other languages were excluded from the review. Duplicated studies were determined by comparing the title of the articles and the authors’ names.

Literature search strategy was conducted on 15th November 2021 via online electronic databases such as Scopus, PubMed and Google Scholar. The keyword inputted for filtering during the search were “post-mortem”, “autopsy” and “COVID-19” in title, abstract and keywords. The data items from the articles were extracted into a spreadsheet consisting of the title of publication, authors and year of publication, the location of study, study design, number of cases, and key findings.

**Fig. 1:**
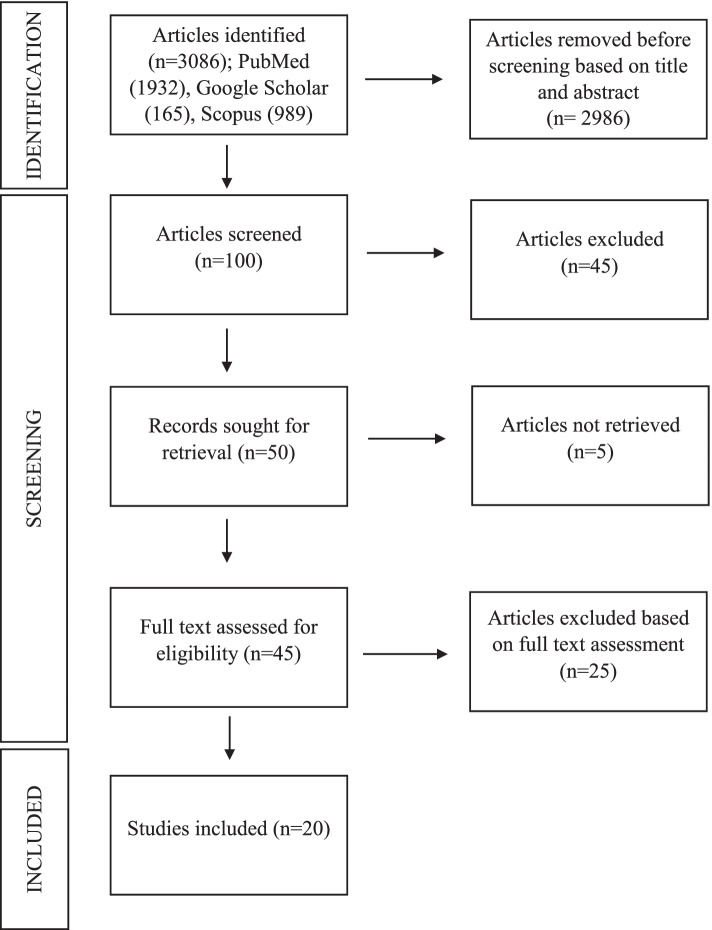
Flowchart of the systematic review process on autopsy findings in COVID-19 deaths following PRISMA model. Footnote: (*n*) refers to number

The titles of the studies were screened and the abstracts were analysed related to the title of this review. Full text was then obtained and studies that fulfilled the inclusion and exclusion criteria were included in the review. The bibliographic references of selected papers were examined to ensure the relevance of those studies. Figure 1 has visualized the flowchart of the reviewing process.

From 3086 articles searched in the above-mentioned databases, 45 full texts were selected after assessment. The bibliography of the selected papers was examined and cross-referenced for further relevant literature. The articles were selected, data were collected and the details were assessed independently by two reviewers so as to mitigate the study risk of bias assessment. These researchers, with medical background, examined the articles and conducted certainty assessment pertaining to the autopsy findings in COVID-19 death. No unpublished and grey literatures were selected. From this approach, a number of 20 relevant papers were included in this review. The rationale of the synthesis methods was monitored by the scientist with systematic review background to ensure the visualisation of the data and tabulation of the data were in a presentable manner. Table 1 was a summary of the systematic review of autopsy findings in COVID-19 death.

**Table 1:** Summary of systematic review of autopsy findings in COVID-19 deaths

Article title	Authors and year of publication	Country of origin	Study design	Number of cases	Summary of findings
A post-mortem examination of COVID-19 pulmonary pathology in 9 cases	Bloom et al. (2020)	USA	Case-control study	9 cases *5 case study patients with severe COVID-19* *4 case-control patients with negative COVID-19*	**Case study**: Microscopic findings on **respiratory system** include - 1) Multifocal to diffuse alveolar necrosis and bronchiolar respiratory epithelial necrosis. 2) Interstitial mononuclear inflammatory infiltrates, mainly lymphocytes, in a multifocal pattern. 3) Perivascular and peribronchiolar lymphoid infiltrates along with marked congestion. 4) Scattered fibroplasia in the severe cases, extending into alveolar spaces and thickening the alveolar septum. 5) Mild hyaline membrane formation and slight microthrombi formations in small pulmonary vasculature found in one case. **Case control**: Characterized by diffuse pulmonary oedema and marked congestion.
Post-mortem examination of COVID-19 patients reveals diffuse alveolar damage with severe capillary congestion and variegated findings in lungs and other organs suggesting vascular dysfunction	Menter et al. (2020)	Switzerland	Autopsy cohort study	21 cases with comorbidity *Hospitalized: median of 7.15 days and mean of 5.7* days	**Respiratory system**: Lungs heterogeneous, ranging from patchy to diffuse areas of consolidation to severe and extensive suppurative bronchopneumonic infiltrate. Mostly present with exudative diffuse alveolar damage (DAD) and 38% with proliferative DAD. **Cardiovascular system**: Myocardial hypertrophy and atherosclerosis at aorta. **Renal system**: signs of shock and acute tubular injury. **Other systems**: Perihilar and peritracheal lymph nodes congested with increased number of plasmablasts.
Pulmonary and systemic involvement in COVID-19 patients assessed with ultrasound-guided minimally invasive autopsy	Duarte-Neto et al. (2020)	Sao Paulo, Brazil	Case series study	10 cases	**Respiratory system**: Exudative-proliferative DAD, fibrinous thrombi in alveolar arterioles and secondary suppurative pneumonia with CD20 positive B cell markers present while proliferative DAD present in longer hospitalized patients. **Other findings**: 1) Related to chronic diseases 2) Finding related to shock 3) Finding unascertained etiologies
The spectrum of histopathologic findings in lungs of patients with fatal COVID-19 infection	Roden et al. (2021)	Rochester, USA	Case study	8 cases with comorbidity: *cardio-vascular disease and dementia*	**Respiratory system**: Lung heavier than normal, patchy consolidation, majority serous pleural effusion, one gross thromboemboli at distal pulmonary artery, chronic inflammation mostly at bronchi and bronchioles, majority present with DAD (acute/organizing/acute and organizing), bronchopneumonia and thromboemboli. CT scan showed extensive mixed ground glass opacities, X-ray showed patchy opacities peripheral and basilar.
Post-mortem lung findings in a patient with asthma and COVID-19	(Konopka et al. 2020)	Michigan, USA	Case report	1 case *(COVID-19 symptoms)*	**Respiratory system**: Heavy edematous lungs with microscopic features of DAD and asthma correlates with history.
Post-mortem findings in Italian patients with COVID-19: a descriptive full autopsy study of cases with and without comorbidities	Falasca et al. (2020)	Rome, Italy	Case report	22 cases	**Respiratory system**: Lungs were heavy, edematous, some have pleural effusion, presence of DAD, cytopathic virus induced changes, thrombi, inflammatory cells infiltrate. **Cardiovascular system**: Hypertrophied heart in all patients with comorbidities and some without, active inflammation (myocarditis and pericariditis). **Renal system**: Interstitial fibrosis and swollen endothelial cells in patients without comorbidities. **Hepatobiliary system**: Sinusoidal congestion & extravasation of red blood cells to space of Disse, microvacuolar and macrovacuolar steatosis. **Other systems**: Lymphoid hypoplasia in spleen with congested red pulp in patients without comorbidities. Microscopically showed replacement of red haematopoietic bone marrow with yellow adipocyte-rich marrow in group 1 patients.
Dying with SARS-CoV-2 infection—an autopsy study of the first consecutive 80 cases in Hamburg, Germany	Edler et al. (2020)	Hamburg, Germany	Case-control study	80 cases *46 males, 34 females* *38% overweight or obese* *Comorbidity: 85% cardio-vascular disease, 55% lung diseases, 35% central nervous system diseases, 21% diabetes mellitus and 16% carcinomas/haematological diseases*	**Cause of Death (COD)**: 57 cases (71%) had pneumonia, with or without evidence of sepsis. Out of ten cases (25%) present with pneumonia, seven cases present with fatal fulminant pulmonary artery embolism while one case each with aortic valve endocarditis, septic encephalopathy, and hepatorenal failure secondary to liver cirrhosis were contributory COD. Total of seven cases (10%) were considered with a competing COD in addition to COVID-19 e.g. aspiration pneumonia, pronounced emphysema without evidence of pneumonia or acute bronchitis. **Respiratory system**: Lung findings include- 1) Broad spectrum of macroscopic changes, often overlaid by chronic diseases such as chronic bronchitis and emphysema. 2) A mosaic-like pattern of pale fields and slightly protruding dark purple sections with prominent capillary drawing seen in COVID-19-associated deaths appeared as a purulent respiratory tract infection with abscessed bronchopneumonia. 3) Microscopically, DAD with activated type II pneumocytes, fibroblasts, protein-rich exudate, and hyaline membranes. In advanced stages, squamous metaplasia and fibrosis occurred. 4) Giant cells and megakaryocytes appeared. The small pulmonary arteries often showed a pronounced infiltrate of lymphocytes and plasma cells, whereby the endothelia were not reactively altered in the sense of vasculitis.
Post-mortem examination of patients with COVID-19	Schaller et al. (2020)	Augsburg, Germany	Case report	12 cases *Comorbidity: Median 4*	**Respiratory system**: Microscopically, DAD present at various stages. **Cardiovascular and hepatobiliary system**: Microscopically, lymphocytic infiltration was seen at myocardium, epicardium and periportal liver.
Pathological study of the COVID-19 through post-mortem core biopsies	Tian et al. (2020)	Wuhan, China	Case report	4 cases	**Respiratory system**: Radiographic findings include bilateral pneumonia with ground glass opacities with/without consolidations. Microscopic findings include features of DAD in all cases with different stages.
COVID-19 Autopsies, Oklahoma, USA	(Barton et al. 2020)	Oklahoma, USA	Case report	2 cases	**Case 1**: 77 years old male presented with underlying hypertension, deep vein thrombosis, splenectomy, osteoarthritis post total knee arthroplasty, and pancreatitis. **COD**: COVID-19 and coronary artery disease. **Respiratory system**: Radiologically, bilateral pulmonary opacities/. Grossly, bilateral lungs heavy and edematous parenchyma. Microscopically, DAD in acute stage, chronic inflammation in bronchi and bronchioles and mucosal edema. **Case 2**: 42 years old male presented with muscular dystrophy. **COD**: Complications of hepatic cirrhosis. **Respiratory system**: Radiologically, bilateral ground glass opacities with consolidations. Grossly, bilateral lungs heavy, red mottled appearance. Microscopically, acute bronchopneumonia with rare, aspirated food particles and no DAD was seen. **Other systems**: Liver cirrhosis with gynecomastia and testicular atrophy were seen.
Histopathology and ultrastructural findings of fatal COVID-19 infections in Washington State: a case series	Bradley et al. (2020)	Washington, USA	Case series	14 cases with comorbidity: *hypertension, chronic kidney disease, obstructive sleep apnea, obesity and diabetes*	**Respiratory system**: Microscopically, DAD presented in acute/organizing phases, and chronic interstitial inflammation. **Other systems**: Other organs presented with comorbidities changes.
Post-mortem examination of hospital inpatient COVID-19 deaths in Lusaka, Zambia - a descriptive whole-body autopsy series	Himwaze et al. (2021)	Lusaka, Zambia	Case study	29 cases with comorbidity: *HIV infection (28%), hypertension (20%), tuberculosis (10%) and diabetes (10%)*	Commonest COD were pulmonary thromboembolism (45%), DAD (31%), and COVID-19 pneumonia (25%). Representative samples were obtained from the various organs (brain, lungs, heart, liver, spleen and kidneys).
COVID-19 autopsies of Istanbul	Arslan et al. (2021)	Istanbul, Turkey	Case study	348 cases	**Respiratory system**: Lung findings include- 1) Sticky gelatinous fluid in cavities, firm and swollen lungs with varying degrees of consolidation were most commonly seen. 2) Microscopically, DAD, type-II pneumocyte hyperplasia, hyaline membrane formation, fibrinous exudate, and fibrinous plaques in the alveoli were the most common findings. 3) Lungs were swollen and tight filled the chest cavity in all cases. 4) Patchy or diffuse interstitial lymphocytic infiltration of viral pneumonia and features of DAD in various stages.
Post-mortem kidney pathology findings in patients with COVID-19	Santoriello et al. (2020)	New York, USA	Cohort study	42 cases *69% males 31% females* *Comorbidity: 73% hypertension, 57% hispanic*	**Renal system:** Kidney findings include- 1) Acute kidney injury (AKI) developed in 31 of 33 patients (94%), including six with AKI stage 1, nine with stage 2, and 16 with stage 3. 2) The predominant finding correlating with AKI was acute tubular injury. 3) Focal kidney fibrin thrombi in six of 42 (14%) autopsies. A single Black patient had collapsing focal segmental glomerulosclerosis (FSGS). 4) Urine dipstick assessment of proteinuria was positive in 23 of 29 subjects (79%) but yielded a urine protein concentration of 100 mg/dl in 76%. 5) Haematuria was present in 19 of 29 individuals, all of whom had indwelling urinary catheters at the time of collection. 6) Hypophosphataemia (17%), Glucosuria (17%), hypokalaemia (6%) were indicators of possible proximal tubular injury.
Post-mortem diagnosis and autopsy findings in SARS-CoV-2 infection: forensic case series	Keresztesi et al. (2020)	Slobozia, Romania	Autopsy case study	15 cases *11 males, 4 females* *Comorbidity: hypertension, coronary artery disease, and history of stroke*	**Respiratory system**: Macroscopically, lungs were congested, firm, heavy lungs with areas of oedematous tissue and patchy involvement, as well as areas of diffuse consolidation. In most cases, the superior airways were mucus free. Microscopically, four cases with bronchopneumonia evidence of DAD with exudative lesions associated with hyaline membranes and oedema. **Renal system**: Kidney injury with acute epithelial tubular necrosis were seen.
Autopsy findings in 32 patients with COVID-19: a single-institution experience	Elsoukkary et al. (2021)	New York, USA	Case study	32 cases	**Main findings**: Total of 27 (84%) patients had macroscopic and/or microscopic thrombi at autopsy. **Respiratory system**: Macroscopic pulmonary thrombi were detected in 11 (34%) cases. Most patients (*n* = 24, 75%) had both exudative and proliferative DAD. Three (9%) patients each showed only acute/exudative DAD or organizing/proliferative DAD. Total of 16 (50%) showed the presence of alveolar neutrophils and 14 (44%) showed evidence of organizing pneumonia. All cases had some degree of type II pneumocyte hyperplasia with reactive atypia and bronchial squamous metaplasia. **Cardiovascular system**: Small intramyocardial vessels contained microthrombi in 6 (19%) and contained fibrin, platelets, or a mixture of both. One case showed acute myocardial infarction. **Other systems**: Thrombi were also observed in the prostatic venous plexus, trachea, lymph nodes, and kidney. Several organs showed concurrent parenchymal infarction.
Pulmonary post-mortem findings in a large series of COVID-19 cases from Northern Italy	Carsana et al. (2020)	Milan, Italy	Case study	38 cases with comorbidity: 18 hypertension, 11 cardio-vascular disorders, 9 diabetes, 4 malignancy, 3 mild chronic obstructive pulmonary disorders	**Respiratory system**: Lung findings include- 1) Exudative and proliferative phases of DAD were found. 2) Capillary congestion, necrosis of pneumocytes, hyaline membrane, interstitial oedema, pneumocyte hyperplasia and reactive atypia with platelet-fibrin thrombi were present. 3) Inflammatory infiltrate was composed by macrophages in alveolar lumens and lymphocytes mainly in the interstice. 4) Electron microscopy revealed viral particles in the cytoplasm of pneumocytes.
A series of COVID-19 autopsies with clinical and pathologic comparisons to both seasonal and pandemic influenza	McMullen et al. (2021)	Chicago, USA	Comparative case series	28 cases *(16 COVID-19 cases, 6 fatal seasonal influenza, 6 fatal pandemic influenza)*	**COVID-19 case findings**: **Respiratory system**: Consolidation (diffuse and focal), haemorrhagic parenchyma, pulmonary oedema, DAD in early to organizing phase, interstitial inflammation, superimposed infections (mostly bacterial) and scattered microscopic thrombi in pulmonary capillaries were discovered. **Other systems**: Thrombotic micro-angiography, myocytes hypertrophy, and acute tubular injury in kidney were seen. **Influenza case findings**: Most cases showed DAD and haemorrhage in respiratory system.
COVID-19 autopsy reports from the Ga-East Municipal and the 37 Military Hospitals in Accra, Ghana	Attoh et al. (2020)	Accra, Ghana	Case report	20 cases with comorbidity *65% diabetes mellitus type 2 or hypertension*	**Respiratory system**: Macroscopically, lungs were heavy, firm, and severely congested. Microscopically, lungs showed features of DAD. **Cardiovascular system**: Heart was enlarged with concentric left ventricle hypertrophy. **Hepatobiliary system**: Liver was heavy with fatty changes and severely congested.
Time to consider histologic pattern of lung injury to treat critically ill patients with COVID-19 infection	(Copin et al. 2020)	Lille, France	Case report	6 cases	**Respiratory system**: Lung findings include- 1) **One case**: Lymphocytic viral pneumonia. 2) **Other five cases**: Acute fibrinous and organizing pneumonia (AFOP) with an extensive intra-alveolar fibrin deposition called fibrin ball, rather than hyaline membrane, was seen. Vascular injury, whereby endothelial injury with cytoplasmic vacuolization and cell detachment in small to medium-sized pulmonary arteries, was also present.

This systematic review has been registered with the National Medical Research Register (NMRR) under the research ID RSCH-ID-22-00025-0IP and the ethical clearance was obtained from the medical research and ethics committee (MREC) with reference to NMRR ID-22-00184-DD8. This systematic review only involved a literature search from the website and did not involve any human subjects or any direct intervention on human subjects. The authors declared that there was no competing interest and no financial support throughout the review process.

## Results

In general, most of the publications were case reports and case series with two case-control study and 1 cohort study. Majority of the articles were published in the year 2020. As for the country of origin, 8 articles originated from the USA, followed by two from Italy, two from Germany and one each from Switzerland, China, Romania, Turkey, Brazil, Zambia, Ghana and France. The lowest number of cases being examined and reported was one whilst the highest was 348 cases. The age of the reported cases varied with the majority of the deceased being elderly. Almost all studies described the majority of the cases have known pre-existing comorbidities with cardiovascular disease and hypertension being the frequently stated risk factors.

### Respiratory system

**Fig. 2:**
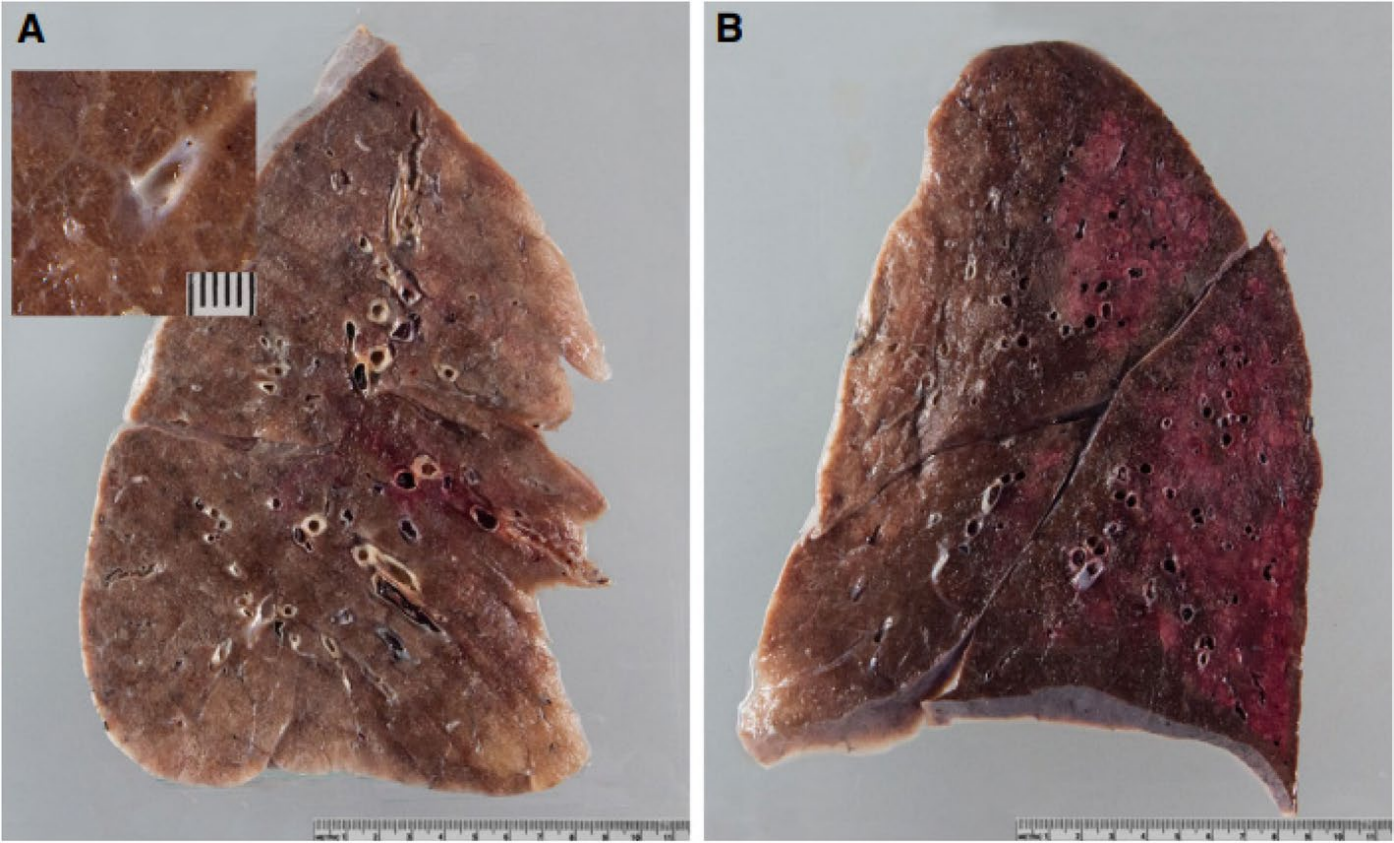
Gross lung findings. **A** Typical appearance of coronavirus disease 2019 (COVID-19) lungs; note the perceptibly thickened alveolar septae and congestive interstitial aspects and a thrombembolus in the lower lobe. Insert: detailed view highlighting interstitial congestion. **B** Extensive bronchopneumonic infiltrates in a COVID-19 patient suffering from superimposed suppurative pneumonia. Note: Source of figure from Menter et al. (2020) (Menter et al. 2020)

Almost all studies reported on the autopsy pulmonary findings in COVID-19 death except for one article reported by Santoriello et al. (2020) that focused on kidney pathology in COVID-19 deaths. Heavy, firm and edematous lungs have been mostly described in (Roden et al. 2021; Konopka et al. 2020; Falasca et al. 2020; Barton et al. 2020; Keresztesi et al. 2020; Attoh et al. 2020) There was patchy to diffuse consolidation in the lungs as mentioned by (Fig. 2).(Roden et al. 2021; Keresztesi et al. 2020; Menter et al. 2020; Arslan et al. 2021)

There were also changes in colour and pattern of the lungs in COVID-19 patients. Edler et al. (2020) described a mosaic-like pattern of pale and dark purple lung sections with prominent capillary drawings. Other than that, Barton et al. (2020) reported a mottled red appearance of the lungs from one of the cases they examined. There was pleural effusion noted in the studies of (Roden et al. 2021; Falasca et al. 2020)

**Fig. 3:**
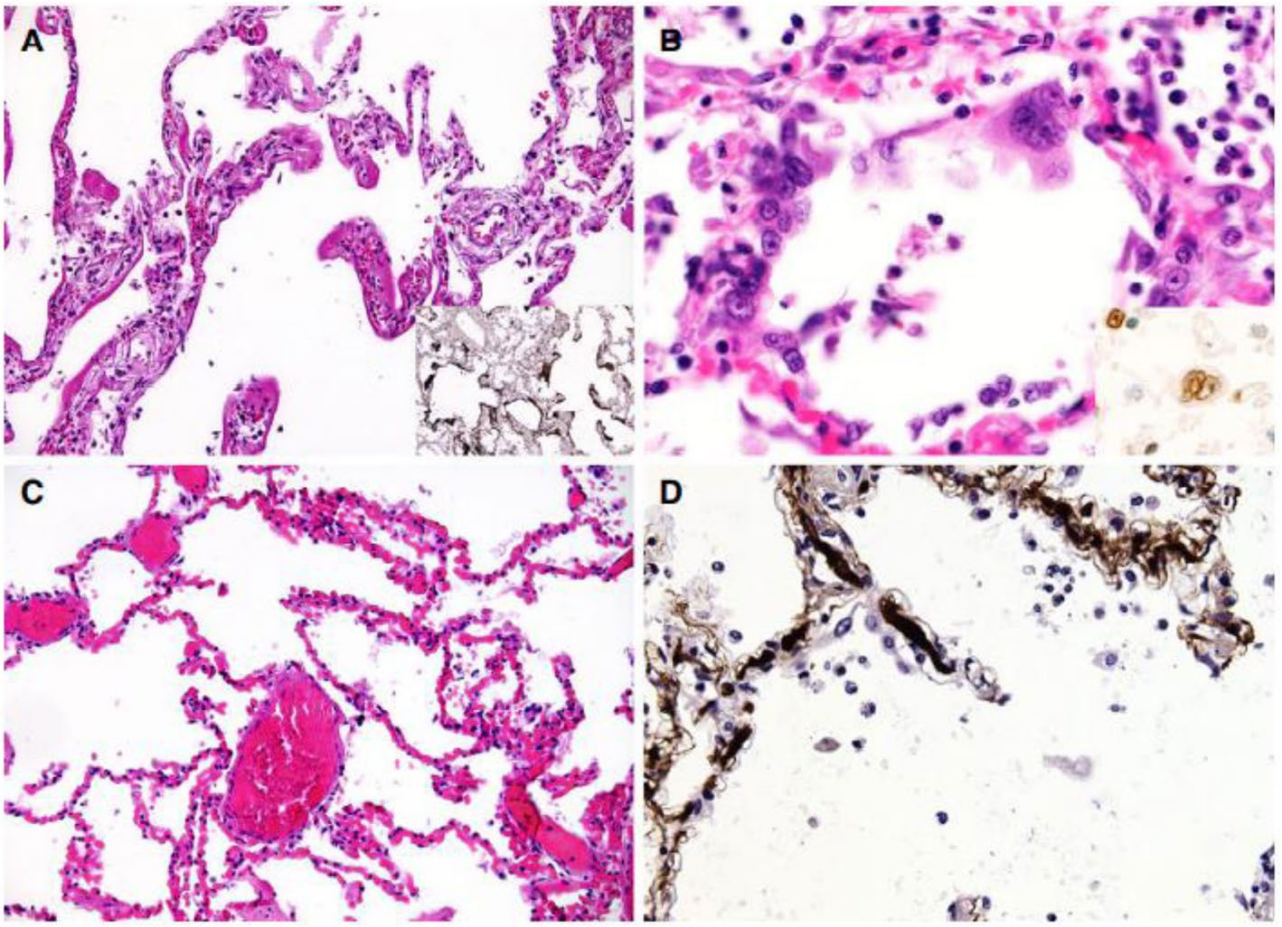
Microscopic lung findings. **A** Exudative diffuse alveolar damage (DAD) showing discrete hyaline membranes and prominent capillary congestion [haematoxylin and eosin (H&E)]. Insert: immunohistochemistry (IHC) for fibrinogen showing the extent of hyaline membranes. **B** Syncytial cells of pneumocyte II origin (H&E). Insert: IHC for thyroid transcription factor 1. **C** Extensive capillary congestion without DAD (H&E). **D** Microthrombi in alveolar capillaries (IHC for fibrin). Note: Source of figure from Menter et al. (2020)

Almost all studies except the study by Santoriello et al. (2020) reported on the histological features of lungs in COVID-19 cases. The most reported finding in all those studies is the features of diffuse alveolar damage (DAD) in the lung histology. Proliferative to exudative diffuse alveolar damage characterized with hyaline membrane deposition, alveolar necrosis, type II pneumocytes hyperplasia and the presence of lymphocytes, macrophages, multinucleated giant cells were considered as distinctive features of COVID-19 infection. Bloom et al. (2020) described the findings as multifocal to diffuse alveolar necrosis and bronchiolar respiratory epithelial necrosis. On the other hand, Edler et al. (2020) reported a diffuse alveolar damage with an accumulation of activated type II pneumocytes, fibroblasts, protein-rich exudate and hyaline membranes. A prominent capillary congestion with hyaline membrane formation, reactive pneumocytes changes and the presence of syncytial cells were the most prominent findings in the study of Menter et al. (2020) (Fig. 3).

There were several studies that described microscopic thrombi formation in the lung histology. Eight out of ten patients in the study of Duarte-Neto et al. (2020) have fibrinous thrombi in alveolar vessels. In addition to that, a study in Northern Italy revealed 33 out of 38 cases have platelet-fibrin thrombi deposited in small alveolar vessels.(Carsana et al. 2020) Elsoukkary et al. (2021) reported 84% of the cases to have microscopic and/or macroscopic thrombi at autopsy and most commonly seen was in the respiratory system. This study also reported the coagulation profile of all patients was prolonged, with the mean prothrombin time of 24 s and mean activated partial thromboplastin time of 51.6 s.

### Cardiovascular system

Pathology of the cardiovascular system was mostly chronic diseases related changes. Duarte-Neto et al. (2020) described the extrapulmonary findings in COVID-19 patients can be categorized into findings related to comorbidities, attributed to shock and unascertained aetiologies. From the study, nine cases were reported with the presence of myocardial hypertrophy and myocardial fibrosis. Myocardial hypertrophy was one of the common findings of the cardiovascular system described in these studies by (Attoh et al. 2020; Menter et al. 2020; Elsoukkary et al. 2021; McMullen et al. 2021) In the study of Menter et al. (2020), 70% of the cases have severe generalized atherosclerosis of the aorta.

Apart from that, there were several studies reported features of cardiac inflammation in the COVID-19 patients. There were two cases of myositis and two cases of mild lymphomononuclear myocarditis in the study of Duarte-Neto et al. (2020). In the study of Falasca et al. (2020), active myocarditis was observed microscopically, characterized by lymphocytic infiltrates and associated with focal myocyte necrosis. There was one case reported by Edler et al. (2020) with a feature of myocarditis such as small lymphocytic infiltrates in the right ventricle of the heart.

### Renal system

The most common finding of the renal system was mainly described as acute tubular injury. Menter et al. (2020) reported features such as widened tubular lumina, flattened tubular epithelium and interstitial oedema which were related to the findings of acute tubular injury. In the study of Santoriello et al. (2020) which more specified towards the kidney pathology in COVID-19 cases, acute tubular injury was the most common finding, especially in patients with acute kidney injury. Acute tubular injury in this study was graded into absent, mild and moderate-to-severe based on the histological changes and its distribution. From their study, they revealed that despite 11 cases that cannot be determined due to autolysis, 39% of the remaining cases were absent or minimal, 39% were mild and 23% were moderate-to-severe.

**Fig. 4:**
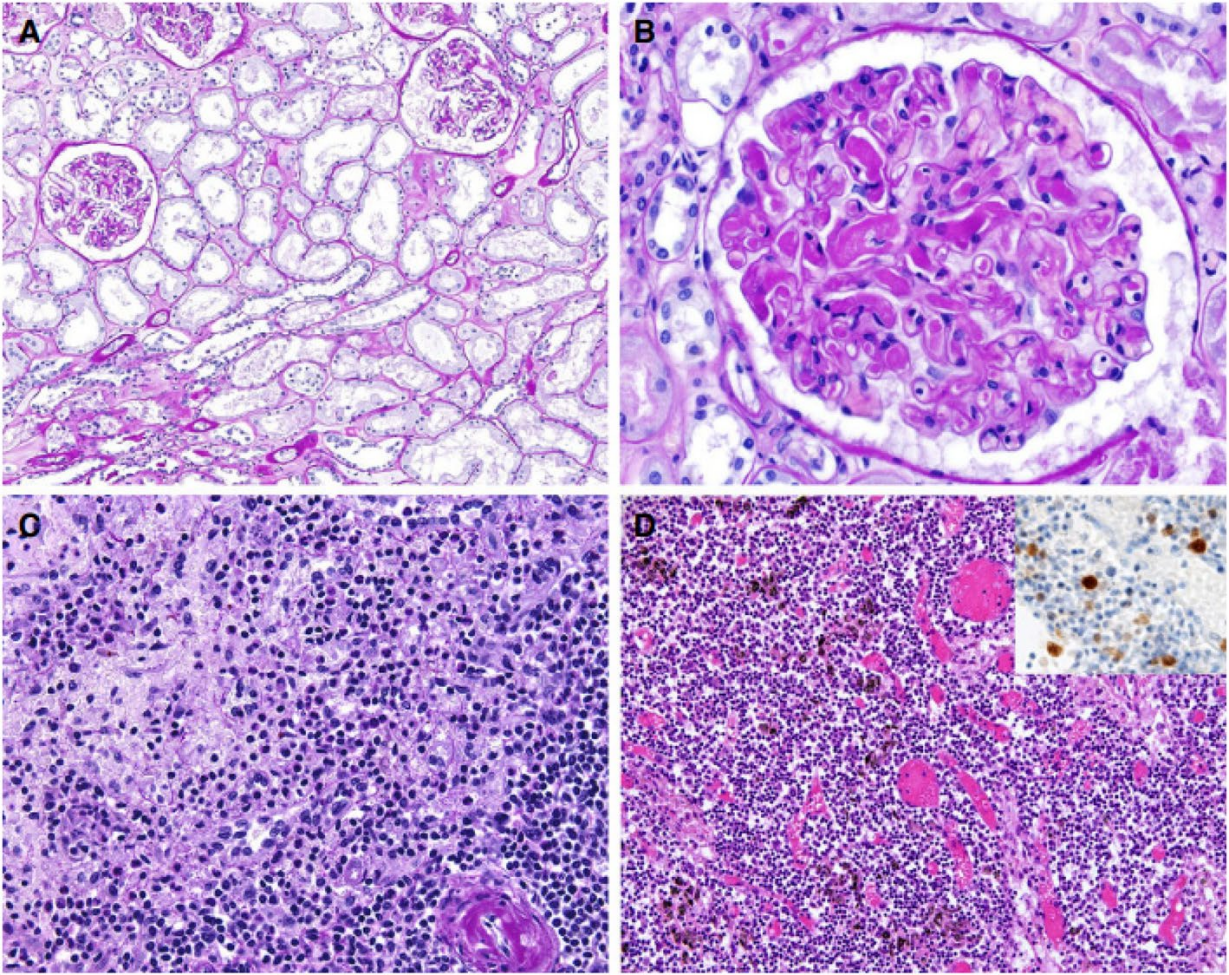
Findings in other organs. **A** Kidney showing acute tubular damage without evidence of increased inflammatory infiltrates [periodic acid–Schiff (PAS) stain]. **B** Kidney showing disseminated intravascular coagulation (PAS). **C** Florid splenitis showing increases in neutrophil numbers in the perifollicular and marginal zones of the spleen (PAS). **D** Lymph node showing an increase in the number of plasmablasts in the interfollicular zone as well as congestion (haematoxylin and eosin). Insert: immunohistochemistry for multiple myeloma 1. Note: Source of figure from Menter et al. (2020)

Other than that, there were also findings related to chronic diseases described in these findings. Chronic changes such as arteriosclerosis, intimal fibrosis of the arteries and vascular scarring were found in the majority of the cases (Fig. 4a, b).(Menter et al. 2020) These findings were also similar to the study of Santoriello et al. (2020) which reported findings such as arteriosclerosis, interstitial fibrosis and tubular atrophy. Such findings also could be found in the study of Duarte-Neto et al. (2020) and Elsoukkary et al. (2021).

### Hepatobiliary and other systems

Congested liver was among the common finding described in these studies of, Falasca et al. (2020), Attoh et al. (2020) and Edler et al. (2020). Other than that, steatosis or fatty changes of liver was also one of the frequently found pathology in these studies by Falasca et al. (2020), Menter et al. (2020) and Elsoukkary et al. (2021). Sign of shock associated necrosis of the liver was described by Menter et al. (2020).

Apart from those liver pathology, haematopoietic system such as spleen and lymph nodes pathology was one of the extrapulmonary organs that often described in these articles (Fig. 4c, d). Elsoukkary et al. (2021) reported in their study that except for one case with splenic marginal zone lymphoma, the lymph nodes were in preserved architecture, morphologically intact germinal centre, paracortical areas and patent sinuses. The subcapsular and intraparenchymal sinuses contained CD20 positive large, transformed cells with prominent nucleoli and amphophilic cytoplasm. These transformed cells were also seen in vascular sinuses and paracortical regions.

## Discussion

### Respiratory system

One of the commonest complications for COVID-19 infections was the involvement of the respiratory system. There were numerous studies that showed lung pathology of the deceased patients who had COVID-19 infection. The lung pathological features of COVID-19 found were similar to severe acute respiratory syndrome (SARS) and the Middle East respiratory syndrome (MERS) (Edler et al. 2020).

In addition to that, some studies show that lung injury was the main cause of death in these patients. The virus was believed to infect the epithelial lining cells of the respiratory tract using the angiotensin-converting enzyme (ACE) 2 enzymes as a viral receptor leading to DAD, edema, and a marked increase in lung weights at autopsy.(Roden et al. 2021; Konopka et al. 2020; Falasca et al. 2020; Barton et al. 2020; Keresztesi et al. 2020; Attoh et al. 2020)

The major histological feature in the lung is diffuse alveolar damage with hyaline membrane formation, alongside microthrombi in small pulmonary vessels. It appears that there is a high incidence of deep vein thrombosis, especially the patients under intensive care despite a prophylactic dose of anticoagulant. Pulmonary embolism and risk for arterial thrombotic disease appear to be increased among COVID-19 decedents, suggesting endothelial involvement, but more studies are needed. (Maiese et al. 2021a; Zanza et al. 2021; Maiese et al. 2020)

Cytokine storm has recently emerged as a key aspect in this disease with affected patients showing high levels of several key pro-inflammatory cytokines, such as IL-1, IL-2, IL-6 and many more. These pro-inflammatory cytokines aggravate interstitial pneumonia and evolve in viral sepsis with prominent hypercoagulability.(Maiese et al. 2020) These agents can be investigated in autopsies cases as part of therapeutic strategies in the near future.(Zanza et al. 2022)

### Cardiovascular system

**Fig. 5:**
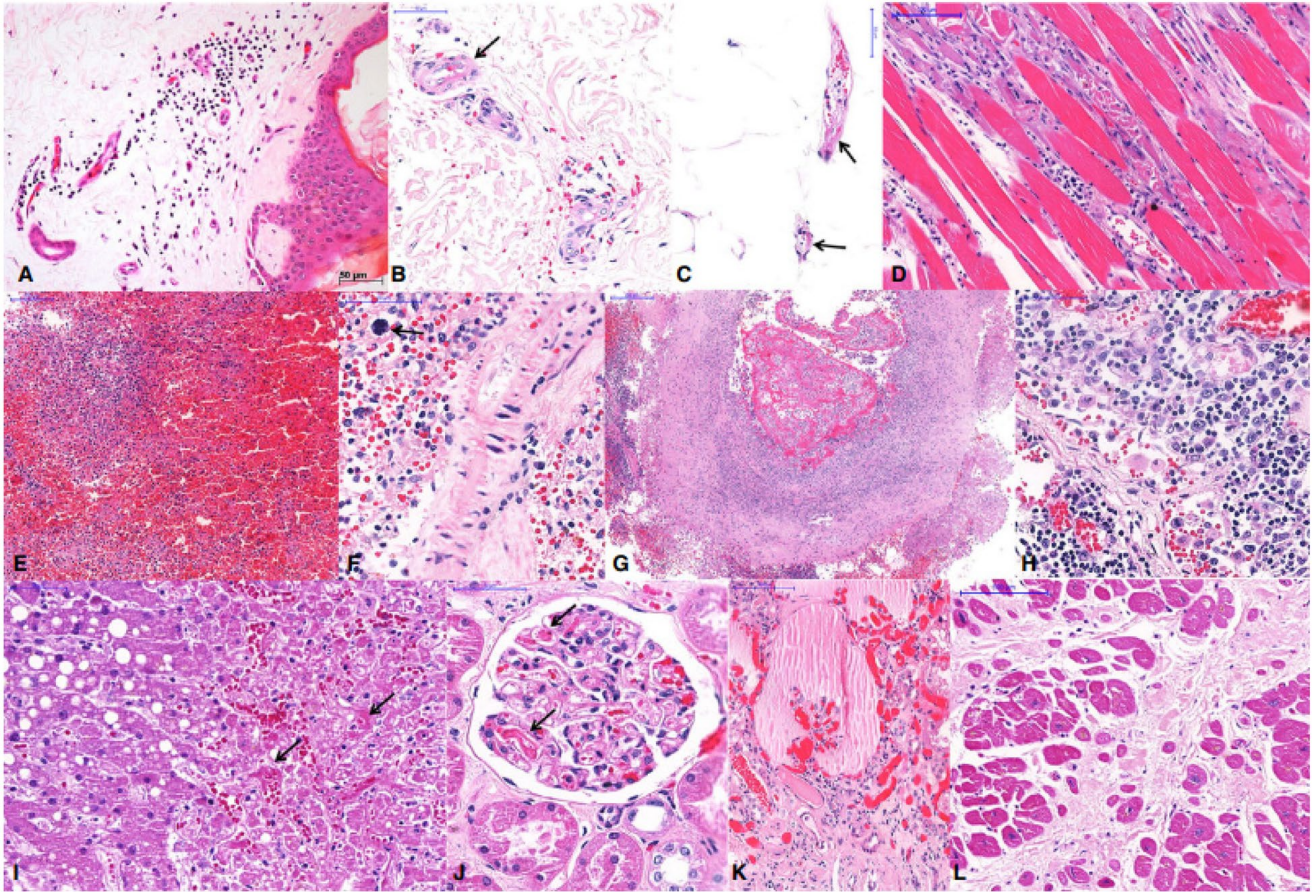
Extrapulmonary histological features of 10 fatal cases of coronavirus disease 19 (COVID-19), autopsied by the use of ultrasound-guided minimally invasive autopsy. **A**–**C** Skin collected with a punch needle, showing a perivascular mononuclear infiltrate at the superficial dermis (**A**), purpura (**B**), and fibrinoid alteration in small vessels of the dermis (**B**) and hypodermis (**C**). **D** Thoracic skeletal muscle with myositis and myolisis. **E**–**G** Spleen showing red pulp haemorrhage and lymphoid hypoplasia (**E**), splenitis and extramedullary haematopoiesis (**F**, arrow), and thrombosis and vasculitis in a large artery (**G**). **H** A thoracic lymph node with hyperplasia of sinusoidal histocytes, haemophagocytosis, and activated lymphocytes. **I** Liver with macrovesicular steatosis, coagulative necrosis in the central area, and sinusoidal congestion with fibrin thrombi (arrows). **J**, **K** Kidney with fibrin thrombi in the capillary tuft (**J**, arrows), and a collapsed tuft and interstitial fibrosis (**K**). **L** Heart with hypertrophy of cardiomyocytes and extensive myocardial fibrosis (previous infarction). Note: Source of figure from Duarte-Neto et al. (2020)

There was some evidence that COVID-19 patients have a significant impact towards their cardiovascular system. Multiple authors have reported clinical evidence of cardiac injury seen in the form of elevated troponins, and pathologically concurrent myocardial infarction and myocarditis.(Falasca et al. 2020) The most significant histologic finding in our cohort was the presence of intramyocardial thrombi. At present, it was known that endothelial injury and thrombosis were seen in patients with COVID-19 infection as a part of microangiopathy.(Elsoukkary et al. 2021) Other than that, ACE2 was highly expressed in the heart, providing opportunity for ACE2-dependent myocardial infarction. Cytokine storm from systemic inflammation and the hypoxic state from acute respiratory distress syndrome (ARDS) inducing excessive extracellular calcium levels leading to myocyte apoptosis were also possible mechanisms of damage. Surge in cytokine levels due to hyperinflammatory response or secondary hemophagocytic lymphohistiocytosis and increased myocardial demand in the setting of acute infection can lead to atherosclerotic plaque instability and myocardial injury, hence increasing the risk of acute myocardial infarction. On the other hand, the finding for gross examination of the heart showed myocardial ventricular hypertrophy and dilatation, mainly of the right cavity, in a considerable number of cases. Acute right coronary artery thrombosis was observed in one case. The most frequent microscopic findings inclusive of cardiomyocyte hypertrophy (Fig. 5l).(Falasca et al. 2020; Duarte-Neto et al. 2020; Elsoukkary et al. 2021)

However, some studies reported that the link of COVID-19 and viral-associated myocarditis does not appear to have a major role in the cardiac injury in these patients. Some of the patients had underlying congenital heart disease requiring multiple surgeries, and the myocarditis was temporally distant, arising earlier than viral symptoms. Therefore, it was believed that this could be unrelated to the viral infection. No other patient had any inflammatory infiltrates in the heart. No viral inclusion was observed in any cases. (Elsoukkary et al. 2021)

Post mortem analysis of the myocyte can be subjected for immunohistochemical examination as there are studies that revealed the constant presence of CD3+ and CD8+ cytotoxic lymphocytes and CD68+ macrophages. COVID-19 leads to a systemic inflammatory response and a constant prothrombotic state.(Maiese et al. 2021b)

### Renal system

In relation to the renal system, some studies mentioned that COVID-19 infection can cause acute tubular injury which may be related to tubular ischaemia alone such as hemodynamic changes or could be further exacerbated by direct viral infection, cytokine mediated injury, or drug-related tubular toxicity. The acute tubular damage was described as flattened tubular epithelium and lumens containing sloughed epithelial lining cells, granular casts, Tamm-Horsfall protein and intraluminal accumulation of cellular debris in focal areas. In addition, considerable renovascular disease was present with moderate-to-severe sclerosis in 80% of cases, atheroemboli, papillary necrosis, and glomerular fibrin thrombi. (Menter et al. 2020)

However, the type of alteration seems to be related to hypoxia and responsible for terminal renal failure, rather than directly associated with the virus, although the tubular epithelium has a high expression of ACE-2 receptors. (Darriverre et al. 2020) Thus, it could be believed that the infection of COVID-19 was indirectly causing damage to the kidney.

### Hepatobiliary and other systems

Liver was one of the affected organs as the infection progresses. Studies showed evidence of liver involvement in the form of steatosis and some cases, necrosis was observed. Apart from steatosis, microscopically it exhibits chronic congestion, lymphocytic infiltrates especially in the portal or periportal tract, hepatocyte necrosis as well as hyperplasia and hypertrophy of the Kupffer cells. The presence of typical coronavirus particles in the cytoplasm, mostly without membrane-bound vesicles, has also been reported. (Falasca et al. 2020)

Involvement of the central nervous system including hypoxic changes, followed by ischemic and haemorrhagic lesions and reactive astrogliosis and microgliosis had been observed. These findings do not seem to be specific to COVID-19 infection but brain examination as part of COVID-19 routine autopsies should be included as part of standardized requirements for further confirmation of the findings. (Maiese et al. 2021c)

## Conclusions

During this pandemic, countless people of various demographics were infected by the COVID-19 virus. However, the most significant group that was hit the worst during this pandemic was those who had previously been diagnosed with comorbidities, hence most of the deceased were older people with pre-existent medical illnesses. The fact that most microscopic findings could be attributed to chronic illnesses and most of the pathology described was universally applied to the older population that suffers from the most common chronic diseases. The currently available information regarding the COVID-19 infection-related death described the systemic target of the infection of mostly respiratory system involvement, followed by cardiovascular, renal and hepatobiliary systems. Autopsy studies allow the researchers to study the flow, pattern and mechanism of the infection that affects the body systems. More research could be done to study the effects of COVID-19 infection on the body's natural defences in order to provide an early warning system and strategies to prevent the infection from becoming fatal. Having said that, there are many issues in relation to future litigation after the COVID-19 pandemic is highlighted especially involving the substandard of care due to various reasons. From the autopsies point of view, the issues of trained personnel, the adequate personal protective equipment as well as the facilities served as the foundation to run a quality autopsy, which might vary from each centre. A uniform COVID-19 post mortem diagnostic protocol has not been developed to date. International collaboration is essential, standardized diagnostic criteria are fundamental requirements as a way forward plan. (Maiese et al. 2021a; Maiese et al. 2021d)

## Data Availability

Not applicable.

## Abbreviations

COVID-19: Coronavirus-19 disease
USA: United States of America
DAD: Diffuse alveolar damage
WHO: World Health Organization
COD: Cause of death
SARS: Severe acute respiratory syndrome
MERS: Middle East respiratory syndrome
ACE: Angiotensin-converting enzyme
ARDS: Acute respiratory distress syndrome

## References

[CR1] Arslan MN, Büyük Y, Ziyade N, Elgörmüş N, Şirin G, Çoban I, Akçay A (2021). COVID-19 autopsies of Istanbul. Irish J Med Sci.

[CR2] Attoh S, Segborwotso RP, Akoriyea SK, Teddy G, Edusei L, Hobenu F, Akakpo PK (2020). COVID-19 autopsy reports from the Ga-east municipal and the 37 military hospitals in Accra, Ghana. Ghana Med J.

[CR3] Barton LM, Duval EJ, Stroberg E, Ghosh S, Mukhopadhyay S (2020). Covid-19 autopsies, Oklahoma, USA. American J Clin Pathol.

[CR4] Bloom A, Colter A, Jacobsen M, Battles D, Albertson T, Sandusky G (2020). A post-mortem examination of COVID-19 pulmonary pathology in 9 cases.

[CR5] Bradley BT, Maioli H, Johnston R, Chaudhry I, Fink SL, Xu H, Marshall DA (2020). Histopathology and ultrastructural findings of fatal COVID-19 infections in Washington state: a case series. Lancet.

[CR6] Carsana L, Sonzogni A, Nasr A, Rossi RS, Pellegrinelli A, Zerbi P, Nebuloni M (2020). Pulmonary post-mortem findings in a series of COVID-19 cases from northern Italy: a two-Centre descriptive study. Lancet Infect Dis.

[CR7] Copin MC, Parmentier E, Duburcq T, Poissy J, Mathieu D (2020). Time to consider histologic pattern of lung injury to treat critically ill patients with COVID-19 infection. Intensive Care Med.

[CR8] Duarte-Neto AN, Monteiro RA, da Silva LF, Malheiros DM, de Oliveira EP, Theodoro-Filho J, Dolhnikoff M (2020). Pulmonary and systemic involvement in COVID-19 patients assessed with ultrasound-guided minimally invasive autopsy. Histopathol.

[CR9] Edler C, Schröder AS, Aepfelbacher M, Fitzek A, Heinemann A, Heinrich F, Sperhake JP (2020). Dying with SARS-CoV-2 infection—an autopsy study of the first consecutive 80 cases in Hamburg, Germany. Int J Legal Med.

[CR10] Elsoukkary SS, Mostyka M, Dillard A, Berman DR, Ma LX, Chadburn A, Salvatore SP (2021). Autopsy findings in 32 patients with COVID-19: a single institution experience. Pathobiol.

[CR11] Falasca L, Nardacci R, Colombo D, Lalle E, Di Caro A, Nicastri E, Del Nonno F (2020). Postmortem findings in Italian patients with COVID-19: a descriptive full autopsy study of cases with and without comorbidities. J Infect Dis.

[CR12] Keresztesi AA, Perde F, Ghita-Nanu A, Radu CC, Negrea M, Keresztesi G (2020). Post-mortem diagnosis and autopsy findings in SARS-CoV-2 infection: forensic case series. Diagnostics.

[CR13] Konopka KE, Wilson A, Myers JL (2020). Postmortem lung findings in a patient with asthma and coronavirus disease 2019. Chest.

[CR14] Maiese A, Passaro G, Matteis A, Raffaele R, Paolo MD (2020). Thromboinflammatory response in SARS-CoV-2 sepsis. Medico-legal J.

[CR15] Maiese A, Frati P, Del Duca F, Turillazzi E, Fineschi V (2021). Myocardial pathology in covid-19-associated cardiac injury: A systematic review. Diagnostics.

[CR16] Maiese A, Manetti AC, Bosetti C, Turillazzi E, Fineschi V (2021). SARS-CoV-2 and the brain: A review of the current knowledge on neuropathology in COVID-19. Brain Pathol.

[CR17] Maiese A, Manetti AC, La Russa R, Frati P, Fineschi V (2021). Autopsy findings in COVID-19-related deaths: a literature review. Forensic Sci Med Pathol.

[CR18] Maiese A, Russa R, Santoro P, Matteis A, Paolo MD (2021). Future litigation after Covid-19 pandemic in Italy. Medico-legal J.

[CR19] Menter T, Haslbauer JD, Nienhold R, Savic S, Hopfer H, Deigendesch N, Tzankov A (2020). Postmortem examination of COVID-19 patients reveals diffuse alveolar damage with severe capillary congestion and variegated findings in lungs and other organs suggesting vascular dysfunction. Histopathol.

[CR20] Roden AC, Bois MC, Johnson TF, Aubry MC, Alexander MP, Hagen CE, Boland JM (2021). The spectrum of histopathologic findings in lungs of patients with fatal coronavirus disease 2019 (COVID-19) infection. Archives Path Lab Med.

[CR21] Santoriello D, Khairallah P, Bomback AS, Xu K, Kudose S, Batal I, Markowitz G (2020). Postmortem kidney pathology findings in patients with COVID-19. J Am Soc Nephrol.

[CR22] Schaller T, Hirschbühl K, Burkhardt K, Braun G, Trepel M, Märkl B, Claus R (2020). Postmortem examination of patients with COVID-19. Jama.

[CR23] Tian S, Xiong Y, Liu H, Niu L, Guo J, Liao M, Xiao SY (2020). Pathological study of the 2019 novel coronavirus disease (COVID-19) through postmortem core biopsies. Mod Pathol.

[CR24] Zaim S, Chong JH, Sankaranarayanan V, Harky A (2020). COVID-19 and multiorgan response. Curr Probl Cardiol.

[CR25] Zanza C, Romenskaya T, Manetti AC, Volonnino G, Longhitano Y (2022). Cytokine storm in COVID-19: Immunopathogenesis and therapy. Medicina (Lithuania).

[CR26] Darriverre L, Fieux F, de la Jonquière C (2020) Acute renal failure during COVID-19 epidemic. Praticien En Anesthesie Reanimation. 10.1016/j.pratan.2020.07.004 10.1016/j.pratan.2020.07.004 PMC7351375 32837207

[CR27] Himwaze CM, Telendiy V, Maate F, Songwe M, Chanda C, Chanda D, Muchelenģanga LA (2021) Post mortem examination of hospital inpatient COVID-19 deaths in Lusaka, Zambia - a descriptive whole body autopsy series. Int J Infect Dis. 10.1016/j.ijid.2021.06.013 10.1016/j.ijid.2021.06.013 PMC8215884 34146690

[CR28] McMullen P, Pytel P, Snyder A, Smith H, Vickery J, Brainer J, Mueller J (2021) A series of COVID-19 autopsies with clinical and pathologic comparisons to both seasonal and pandemic influenza. J Pathol Clin Res. 10.1002/cjp2.220 10.1002/cjp2.220 PMC8239851 33960723

[CR29] Mokhtari T, Hassani F, Ghaffari N, Ebrahimi B, Yarahmadi A, Hassanzadeh G (2020) COVID-19 and multiorgan failure: a narrative review on potential mechanisms. J Mol Histol:1–16. 10.1007/s10735-020-09915-3 10.1007/s10735-020-09915-3 PMC7533045 33011887

[CR30] World Health Organization (WHO). COVID-19 strategic preparedness and response plan: operational planning guideline (1 February 2021 to 31 January 2022). https://apps.who.int/iris/handle/10665/340073. Accessed 30 Nov 2021.

[CR31] World Health Organization (WHO). COVID-19 weekly epidemiological update (edition 68). https://apps.who.int/iris/handle/10665/350006. Accessed 30 Nov 2021.

[CR32] Zanza C, Racca F, Longhitano Y, Volonnino G, Russa RL (2021) Risk management and treatment of coagulation disorders related to COVID-19 infection. Int J Environ Res Public Health 18(3)1. 10.3390/ijerph18031268 10.3390/ijerph18031268 PMC7908596 33572570

